# Convergent Evolution Driven by Rifampin Exacerbates the Global Burden of Drug-Resistant *Staphylococcus aureus*

**DOI:** 10.1128/mSphere.00550-17

**Published:** 2018-01-24

**Authors:** Romain Guérillot, Anders Gonçalves da Silva, Ian Monk, Stefano Giulieri, Takehiro Tomita, Eloise Alison, Jessica Porter, Sacha Pidot, Wei Gao, Anton Y. Peleg, Torsten Seemann, Timothy P. Stinear, Benjamin P. Howden

**Affiliations:** a Department of Microbiology and Immunology, The University of Melbourne at the Doherty Institute for Infection and Immunity, Melbourne, Victoria, Australia; b Doherty Applied Microbial Genomics, The University of Melbourne at the Peter Doherty Institute for Infection and Immunity, Melbourne, Victoria, Australia; c Microbiological Diagnostic Unit Public Health Laboratory, The University of Melbourne at the Peter Doherty Institute for Infection and Immunity, Melbourne, Victoria, Australia; d Biomedicine Discovery Institute, Department of Microbiology, Faculty of Medicine, Nursing and Health Sciences, Monash University, Clayton, Victoria, Australia; e Department of Infectious Diseases, The Alfred Hospital and Central Clinical School, Monash University, Melbourne, Victoria, Australia; f Melbourne Bioinformatics, University of Melbourne, Melbourne, Victoria, Australia; g Infectious Diseases Department, Austin Health, Heidelberg, Victoria, Australia; University of Nebraska Medical Center

**Keywords:** *Staphylococcus aureus*, adaptive mutations, adaptive resistance, antibiotic resistance, drug resistance evolution, genomics, vancomycin

## Abstract

Mutations in the beta-subunit of bacterial RNA polymerase (RpoB) cause resistance to rifampin (Rif^r^), a critical antibiotic for treatment of multidrug-resistant *Staphylococcus aureus*. *In vitro* studies have shown that RpoB mutations confer decreased susceptibility to other antibiotics, but the clinical relevance is unknown. Here, by analyzing 7,099 *S. aureus* genomes, we demonstrate that the most prevalent RpoB mutations promote clinically relevant phenotypic plasticity resulting in the emergence of stable *S. aureus* lineages, associated with increased risk of therapeutic failure through generation of small-colony variants (SCVs) and coresistance to last-line antimicrobial agents. We found eight RpoB mutations that accounted for 93% (469/505) of the total number of Rif^r^ mutations. The most frequently selected amino acid substitutions affecting residue 481 (H481N/Y) were associated with worldwide expansions of Rif^r^ clones spanning decades. Recreating the H481N/Y mutations confirmed no impact on *S. aureus* growth, but the H481N mutation promoted the emergence of a subpopulation of stable Rif^r^ SCVs with reduced susceptibility to vancomycin and daptomycin. Recreating the other frequent RpoB mutations showed similar impacts on resistance to these last-line agents. We found that 86% of all Rif^r^ isolates in our global sample carried the mutations promoting cross-resistance to vancomycin and 52% to both vancomycin and daptomycin. As four of the most frequent RpoB mutations confer only low-level Rif^r^, equal to or below some international breakpoints, we recommend decreasing these breakpoints and reconsidering the appropriate use of rifampin to reduce the fixation and spread of these clinically deleterious mutations.

**IMPORTANCE** Increasing antibiotic resistance in the major human pathogen *Staphylococcus aureus* is threatening the ability to treat patients with these infections. Recent laboratory studies suggest that mutations in the gene commonly associated with rifampin resistance may also impact susceptibility to other last-line antibiotics in *S. aureus*; however, the overall frequency and clinical impact of these mutations are unknown. By mining a global collection of clinical *S. aureus* genomes and by mutagenesis experiments, this work reveals that common rifampin-induced *rpoB* mutations promote phenotypic plasticity that has led to the global emergence of stable, multidrug-resistant *S. aureus* lineages that are associated with increased risk of therapeutic failure through coresistance to other last-line antimicrobials. We recommend decreasing susceptibility breakpoints for rifampin to allow phenotypic detection of critical *rpoB* mutations conferring low resistance to rifampin and reconsidering the appropriate use of rifampin to reduce the fixation and spread of these deleterious mutations globally.

## Abstract (precis)

Increasing antibiotic resistance in the major human pathogen *Staphylococcus aureus* is threatening the ability to treat patients with these infections. Recent laboratory studies suggest that mutations in the gene commonly associated with rifampin resistance may also impact susceptibility to other last-line antibiotics in *S. aureus*; however, the overall frequency and clinical impact of these mutations are unknown. By mining a global collection of clinical *S. aureus* genomes and by mutagenesis experiments, this work reveals that common rifampin-induced *rpoB* mutations promote phenotypic plasticity that has led to the global emergence of stable, multidrug-resistant *S. aureus* lineages that are associated with increased risk of therapeutic failure through coresistance to other last-line antimicrobials. We recommend decreasing susceptibility breakpoints for rifampin to allow phenotypic detection of critical *rpoB* mutations conferring low resistance to rifampin and reconsidering the appropriate use of rifampin to reduce the fixation and spread of these deleterious mutations globally.

## INTRODUCTION

*Staphylococcus aureus* is a major opportunistic human pathogen that causes a wide range of diseases, including endocarditis, sepsis, and pneumonia (1, 2). Its potential to cause hospital outbreaks and community-acquired infections is well recognized, and increasing antibiotic resistance, especially methicillin-resistant *S. aureus* (MRSA), is considered a global public health threat (3). Since its introduction more than 50 years ago, vancomycin has been the treatment of choice for invasive infection with MRSA. However, the efficacy of vancomycin has decreased over the past 2 decades with the emergence of vancomycin-intermediate *S. aureus* (VISA), heterogeneous VISA (hVISA), and to a lesser extent, high-level vancomycin-resistant *S. aureus* (VRSA) (4, 5).

Rifampin is one of a small number of antimicrobial agents that retains activity against multidrug-resistant MRSA. Rifampin has potent antistaphylococcal activity and is currently indicated in combination therapy for implant-associated infections, serious *S. aureus* infections, and to eradicate asymptomatic carriage of MRSA (6, 7). There are increasing reports of combination therapy with rifampin, but surprisingly, there is little documented evidence to support its use (7–9). The effectiveness of coadministration of rifampin with other agents to treat invasive and persistent *S. aureus* infection and to eradicate colonization are currently being evaluated by several large clinical trials (10, 11).

Bacterial resistance to rifampin (Rif^r^) arises from mutations that alter residues of the rifampin binding site of the RNA polymerase (12). Rif^r^ mutations have been almost exclusively located in the *rpoB* gene that encodes the β subunit of the RNA polymerase in a cluster forming the Rif^r^-determining region (RRDR). As rifampin-induced mutations affect the central machinery responsible for gene expression in bacteria, resistance selection can cause pleiotropic effects by modifying the transcription profile and kinetics of the bacterial cell (13–15).

In *S. aureus*, a number of studies have revealed a worrying link between certain *rpoB* mutations and decreased susceptibility not only to rifampin but also to other last-line anti-MRSA antibiotics such as vancomycin, daptomycin, beta-lactams, or imipenem (16–19). Most of these resistance mutations have been generated and assessed by *in vitro* selection experiments, and the clinical relevance of these changes is uncertain. The link between *rpoB* mutations and reduced vancomycin susceptibility is especially concerning, as rifampin and vancomycin are often coadministered, despite a lack of evidence supporting this practice. There is also growing evidence of poorer therapeutic outcome to persistent MRSA infections associated with reduced vancomycin susceptibility (4, 20–22).

Mutations affecting *rpoB* are frequently isolated from hVISA and VISA clinical isolates (16, 23), and the amino acid residue at position 481 of RpoB has recently been found to be the strongest genetic marker of increased vancomycin resistance in two genome-wide association studies (24, 25). A new type of vancomycin resistance, called slow-intermediate vancomycin-resistant *S. aureus* (sVISA) has been recently identified *in vitro* and linked to *rpoB* mutations (26). This phenotype is associated with a pinpoint colony morphology with a very slow doubling time that displays levels of resistance to vancomycin equal to or greater than those of VISA (≥6 mg/liter). These colonies can quickly revert to the wild-type phenotype with lower vancomycin MIC. From this observation, it has been proposed that *rpoB* mutations represent regulatory mutations that serve to switch vancomycin resistance on and off. The broader negative clinical consequences of *S. aureus rpoB* mutations were recently suggested when the resistance phenotype conferred by *rpoB* mutations also promoted immune evasion and modulation of virulence factors (15).

An extensive review of the literature identified 29 scientific articles referring to a total of 89 different *S. aureus rpoB* alleles (see Table S1 in the supplemental material). The majority (77.5% [69/89]) of the *rpoB* mutations reported were identified after *in vitro* selection (predominately to rifampin, but also to vancomycin, daptomycin, beta-lactams and co-trimoxazole); of these mutations, 22 were identified from both *in vitro* selection and in clinical isolates. Significantly, however, there were 20 mutations identified in clinical isolates only, suggesting that *in vitro* experiments do not capture the full breadth of conditions to which *S. aureus* bacteria are exposed *in vivo* (Table S1).

10.1128/mSphere.00550-17.3 TABLE S1  Literature review of phenotypes associated with RpoB mutations. Download TABLE S1, XLS file, 0.1 MB. Copyright © 2018 Guérillot et al. 2018 Guérillot et al. This content is distributed under the terms of the Creative Commons Attribution 4.0 International license.

Intriguingly, three clinical Rif^r^ mutations have been associated with different impacts on pathogenesis. The Rif^r^ mutation *rpoB*-H481Y (the H-to-Y change at position 481 encoded by *rpoB*) acquired during rifampin treatment has been found to associate with persistent infection by both attenuating host innate immune responses and promoting cross-resistance to vancomycin and daptomycin (15, 18). The mutation *rpoB*-A477D that arose *in vivo* after daptomycin treatment was found to confer a range of phenotypes, including increased cell wall thickness, reduced expression of virulence traits, induced expression of the stress-associated transcriptional regulator Spx, and slow growth in addition to decreased susceptibility to daptomycin, vancomycin, β-lactams, and rifampin (27). The Rif^r^ mutation *rpoB*-S464P was found to provide a competitive advantage over the rifampin-susceptible parent strain in a mouse biofilm infection model (28).

Thus, not all *rpoB* mutations have the same phenotypic consequences, and there are potentially different clinical impacts for patients (15–19, 26, 27). Because the majority of studies that have examined *rpoB* mutations rely on mutants generated by *in vitro* rifampin exposure, these mutations might not be the same as those that arise *in vivo*, where there are more-complex selective pressures that include exposure to multiple antibiotics and host immune responses. Thus, while it is well recognized that mutations in *rpoB* play a central role in *S. aureus* antimicrobial resistance and persistence, we lack an accurate understanding of the commonly selected mutations among *S. aureus* globally and the phenotypic consequences linked to specific mutations.

In this study, we aim to address this issue by interrogating thousands of publically available *S. aureus* genome sequences to evaluate the clinical significance of *rpoB* mutation acquisition in this pathogen. By identifying preferentially selected and fixed *rpoB* mutations and then reconstructing these mutations in MRSA, we show that increased rifampin usage and resistance are having far broader and more-dramatic negative clinical consequences than previously recognized.

## RESULTS

### Comparative analysis of *rpoB* gene sequences among 7,099 *S. aureus* genomes.

**FIG 1:**
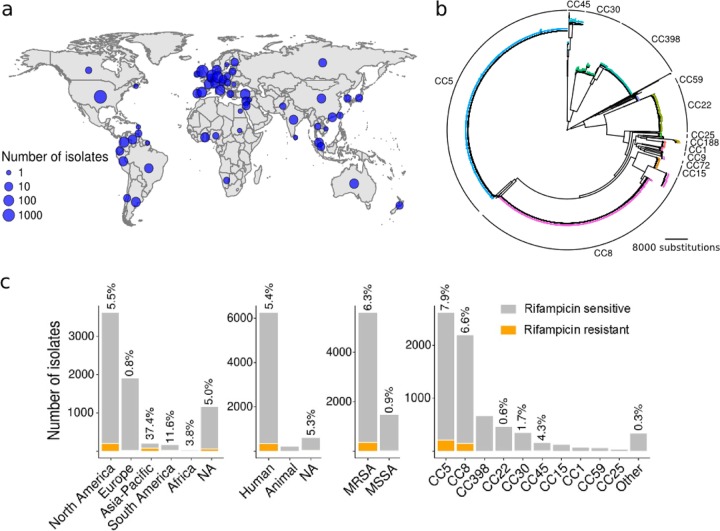
Origin and diversity of the 7,099 sequenced strains. (a) Geographical distribution of the analyzed isolates. (b) Global core genome SNP phylogeny of the 7,099 isolates analyzed. Major clonal complexes are annotated by colored tip points. (c) Origin and frequencies of predicted Rif^r^ among isolates. Each bar represents the total number of isolates, and orange bars represent the number of predicted Rif^r^ isolates. The percentages at the top of the bars represent the percentages of predicted Rif^r^ isolates. NA, not available.

As a first step to better understand the full impact of *rpoB* mutations, we compared *rpoB* gene sequences from 7,099 publicly available *S. aureus* genomes. The genomes came from isolates spanning 58 countries across all continents except Antarctica (Fig. 1a). Phylogenetic inference generated from core genome single nucleotide polymorphisms (SNPs) highlighted the genetic diversity of *S. aureus*, with a highly structured population of dominant clonal complexes (CCs) (Fig. 1b). *In silico* determination of isolate sequence types (STs) showed broad genetic diversity, with 165 different STs corresponding to 37 clonal complexes, with clonal complex 5 (CC5) and CC8 being the dominant clonal complexes (Fig. 1c). The majority of isolates came from North America (57%) and Europe (27%), and most isolates were of human origin (80%) and were predicted to be MRSA (79%) (Fig. 1c).

**FIG 2:**
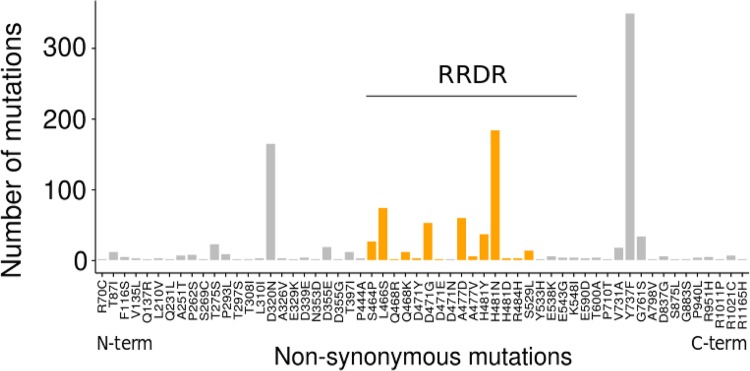
Occurrence and positions of RpoB amino acid substitutions. RpoB nonsynonymous amino acid substitutions identified among the 7,099 genomes are shown. Amino acid substitutions previously associated with Rif resistance are indicated by orange bars. Only mutations observed in three or more isolates are plotted. Amino acid substitutions are shown from left to right from the most N-terminal residues to the most C-terminal residues of RpoB. All RpoB nonsynonymous substitutions are identified in the rifampin resistance-determining region (RRDR).

Among the 7,099 genomes, we found 505 different *rpoB* alleles encoding 245 different RpoB proteins. We observed high amino acid sequence conservation with 83% of the isolates sharing identical RpoB protein sequences. In total, 233 different *rpoB* nonsynonymous mutations were identified comprising 232 amino acid substitutions and a single duplication (Q468). The most prevalent nonsynonymous *rpoB* mutations are presented in Fig. 2. Among the 15 most frequent *rpoB* mutations, 8 corresponded to previously identified Rif^r^ mutations (Fig. 2 and see Table S1 in the supplemental material). The most prevalent RpoB mutation conferring Rif^r^ was H481N observed in 185 strains (2.6%). The first and third most frequent mutations, Y737F and D320N, respectively, do not confer Rif^r^ (29). These mutations may represent genetic drift or reflect a selective pressure other than rifampin (see positive convergent evolution of Rif^r^ mutations below).

Among the 1,184 amino acid residues of RpoB, 196 positions (16.5%) were mutated at least once. All previously recognized mutations conferring Rif^r^, based on earlier descriptions (Table S1), were identified in a single cluster of the RRDR from residues 464 to 529 (Fig. 2). There were no Rif^r^ mutations in the N-terminal or C-terminal extremities of RpoB, unlike what has been reported for *in vitro*-selected *S. aureus* rifampin-resistant mutants (30) or in other bacteria, such as *Mycobacterium tuberculosis* (31) and *Escherichia coli* (32).

### Prediction of rifampin resistance among sequenced genomes.

We assessed the distribution of previously identified Rif^r^ mutations (Table S1) among the 7,099 isolates. In total, 370 isolates carried a Rif^r^-associated *rpoB* allele and were predicted to be resistant to rifampin (5.2% of the total number of isolates). The proportion of predicted Rif^r^ isolates differed with respect to isolate origin. *S. aureus* isolates from Europe contained fewer predicted Rif^r^ mutations (0.8%), which is in accordance with previous Rif^r^ studies and European Centre for Disease Prevention and Control (ECDC) reports (33). The Asia-Pacific region and South America had high levels of predicted Rif^r^ (37% and 11.6%, respectively). Of the North American isolates, 5.5% were predicted to be Rif^r^, close to the global prevalence (Fig. 1b).

The proportion of predicted Rif^r^ isolates was significantly higher among MRSA compared to methicillin-sensitive *S. aureus* (MSSA) (6.7% versus 0.9%). Rif^r^ was predicted in six different CCs and 18 STs. CC5 and CC8 have the highest rates of Rif^r^ (7.9% and 6.6%, respectively); however, Rif^r^ isolates were identified among all major CCs (CC5, CC8, CC22, CC30, and CC45), with the exception of the livestock-associated CC398 (0/669). Interestingly, no Rif^r^ strains were predicted among the 221 animal strains analyzed, suggesting that Rif^r^ selection is not associated with veterinary use. This observation can be linked to the fact that rifampin is not specifically approved for veterinary use in North America, and its use in veterinary medicine is limited in Europe (according to the American Academy of Veterinary Pharmacology and Therapeutics and the European Medicine Agency). Several STs, such as ST5, ST105, ST239, and ST609, had high frequencies of Rif^r^ isolates (6.7%, 8.5%, 76%, and 14.3%, respectively), which suggests that expansion of Rif^r^ clones has occurred (see below).

### Mutations in RpoB conferring resistance to antibiotics other than rifampin identified by *in vitro* experiments are extremely rare in clinical isolates.

As several *rpoB* mutations identified outside the RRDR and not conferring Rif^r^ have been associated *in vitro* with increased resistance to different last-line antibiotics (Table S1), we screened our large genomic database for the presence of those mutations. Among the 29 mutations that have been associated with an increase in vancomycin MIC after *in vitro* exposure, only one non-Rif^r^ mutation, R406S, was observed in a single isolate. We did not identify any mutations associated with the newly described slow-VISA phenotype associated with low growth rate and high vancomycin MIC (26). None of the *in vitro*-selected mutations R512P, G744R, S746F, H929T, and G977V were found. Similarly, none of the mutations selected with daptomycin (six mutations), oxacillin (two mutations), and imipenem and co-trimoxazole were found among the 7,099 genomes, raising questions about the clinical significance of these mutations identified after *in vitro* selection. In contrast, Rif^r^-selected mutations that have been associated with reduced susceptibility to other antibiotics (cross-resistance phenotype) were abundant, found in 165 of the 370 Rif^r^ isolates.

### Convergent evolution of Rif^r^ mutations.

**FIG 3:**
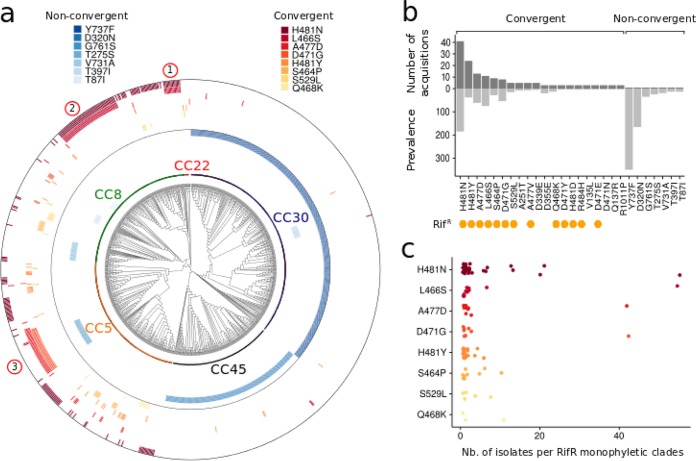
Phylogenetic and convergence analyses of *rpoB* mutations. (a) Reduced core genome phylogeny of the most prevalent RpoB substitutions. The tree represents the phylogenetic relationships of a subset of the 7,099 isolates. Only isolates carrying the most common RpoB substitutions (occurrence > 10) were kept. For each monophyletic clade sharing an identical RpoB substitution, the closest related isolates showing an absence of this mutation were also kept in the phylogeny in order to show cases of independent acquisition of the same RpoB mutation (convergence). The red outer heatmap shows RpoB substitutions associated with multiple acquisition/convergence events indicative of positive selection. The blue inner heatmap shows RpoB substitutions associated with single acquisition/convergence events indicative of genetic drift. The numbers circled in red indicate the positions of the monophyletic clades sharing the same RpoB substitution that are represented in Fig. 4. (b) Inferred number of independent acquisitions and prevalence of most common RpoB mutations. The dark gray bars represent the inferred number of independent acquisitions of most common RpoB mutations. The light gray bars represent the number of isolates identified with these mutations. Previously identified Rif^r^ mutations are indicated by orange circles below the mutations on the *x* axis. (c) Sizes of monophyletic clades with identical RpoB mutations. Each circle represents a monophyletic clade sharing an identical RpoB mutation. The number of identified isolates per monophyletic clade (size of clades) is plotted on the *x* axis. The different types of RpoB mutations are plotted on the *y* axis and are represented by different shades of red.

We performed large-scale evolutionary convergence analysis of all *rpoB* mutations by looking for independent mutation acquisition along the inferred phylogeny of the 7,099 isolates (34, 35). Using this approach, we found 32 convergent mutations among the 233 different RpoB mutations (Table S2). Of these mutations, 16 changes represent known Rif^r^ mutations, which in turn represent 74% of convergent mutation acquisition events (137/186). The seven most frequently selected mutations comprised only Rif^r^ mutations (106 selection events) (Fig. 3b). A simplified phylogeny in Fig. 3a represents the convergent or nonconvergent state of the most prevalent *rpoB* mutations. We also counted the inferred number of independent acquisitions of convergent *rpoB* mutations (Fig. 3b and Table S2). The most frequently observed Rif^r^ mutation, H481N, appeared to also be the most likely to be independently acquired, suggesting that this mutation is the most likely to emerge under rifampin selective pressure *in vivo*. Interestingly, the second most selected mutation, H481Y, affects the same *rpoB* residue and has been associated with persistent bacterial infections, immune evasion, and small-colony variant (SCV) phenotype (15, 36). We also identified unknown *rpoB* mutations that have been found to be independently acquired three or more times: A251T, D339E, D355E, V135L, D471N, Q137R, and R1011P (Fig. 3b and Table S2). Two of these changes, both identified in three isolates only, affect residues that have already been implicated in Rif^r^ (D471 and Q137 [Table S1]), and therefore likely represent new, rare Rif^r^ mutations.

10.1128/mSphere.00550-17.4 TABLE S2  Convergence state and prevalence of all nonsynonymous RpoB mutations. Download TABLE S2, XLS file, 0.1 MB. Copyright © 2018 Guérillot et al. 2018 Guérillot et al. This content is distributed under the terms of the Creative Commons Attribution 4.0 International license.

Convergence analysis also determined that the two dominant mutations Y737F and D320N are found in large monophyletic clades, resulting from single events of mutation acquisition (Fig. 3a). The absence of evolutionary convergence signatures for these mutations strongly suggests that they did not emerge because of evolutionary selective pressure. Similarly, we determined that the frequent *rpoB* mutations G761S, T275S, V731A, T397I, T87I, and P262S can be solely explained by genetic drift and not selection (Fig. 3a and Table S2). Taking all the results together, we show here that rifampin is the major selective pressure driving *rpoB* evolution and that large-scale convergence analysis can be effectively applied to identify and discriminate resistance mutations from high-frequency mutations resulting from genetic drift.

### Phylogenetic persistence of rifampin resistance mutation.

**FIG 4:**
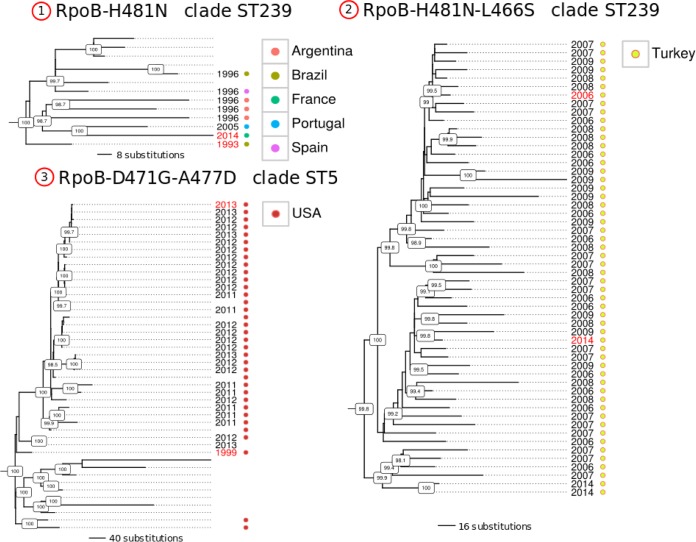
Successful spread and transmission of rifampin-resistant clones. Each tree represents phylogenetic relationships of monophyletic clades sharing identical Rif^r^ mutations. Boxed numbers on nodes represent (Shimodaira-Hasegawa test [SH]-like) branch support values inferred by FastTree. When available, the isolation date (year) of the strains is shown to the right of the tree. For each tree, the youngest and oldest isolation date labels are colored in red. Different colored dots represent the different isolation countries of the strains according to the color keys.

We then investigated whether specific Rif^r^ mutations were more likely to be associated with successful clonal expansion than others. We counted the number of clones for each monophyletic clade corresponding to a single Rif^r^ mutation acquisition event. The number of isolates identified within each Rif^r^ clade is presented in Fig. 3c. Strikingly, we identified numerous large monophyletic clades carrying the same Rif^r^ mutation, suggesting successful spread of rifampin-resistant clones once the mutation emerges. These clades showed strong branch support in the context of the 7,099 isolates and remained monophyletic when using different maximum likelihood tree optimization methods or by inferring trees from CC-specific core genome alignment. The H481N, L466S, D471G, and A477D mutations were associated with the largest clades with up to 54 isolates for one clade sharing the H481N and L466S mutations. Because these publicly available *S. aureus* genomes may have been deposited as part of single study or outbreak investigations where local clonal dissemination explained the large number of resistant isolates, we then searched for available metadata information about the year and country of isolation associated with each clade. We found that different Rif^r^ clones appear to have persisted for decades and/or were associated with worldwide dissemination. Examples of such evolutionarily successful rifampin-resistant clones are presented in Fig. 4. Clonal expansion over decades and international dissemination of some Rif^r^ clones demonstrate that some Rif^r^* rpoB* mutations do not impair transmission and global dissemination. Importantly, they also demonstrate that our conclusions are not likely to be significantly affected by sampling biases in the international database of *S. aureus* genomes.

### A restricted subset of Rif^r^ mutations are preferentially selected among clinical isolates.

Phylogenetic analysis revealed that some *rpoB* Rif^r^ mutation acquisitions are repeatedly associated with successful clonal expansion and dissemination of persistent clones. These observations suggest that these particular Rif^r^ mutations are favored. To investigate selective bias among the repertoire of Rif^r^ mutations, we first compared the mutations identified *in vitro* to the mutations observed in human isolates. Among the repertoire of 46 Rif^r^* rpoB* alleles identified *in vitro*, more than half (24/46) were never observed in our data set of 7,099 genomes, suggesting differences in selection pressure *in vivo*. We then examined the predictive power for Rif^r^ of the 22 different mutations identified among our collection. We found that the eight most common mutations are sufficient to predict 92% (342/370) of clinical Rif^r^ strains (at least one mutation conferring Rif^r^) and that they represent 93% (469/505) of the total number of detected Rif^r^ mutations (Fig. S1). Furthermore, along the evolutionary history of these 7,099 isolates, these eight mutations represent 79% (114/186) of the inferred number of selection events leading to Rif^r^. Taken together, these observations indicate that selection and fixation of Rif^r^ at the molecular level are highly skewed toward a small subset of specific *rpoB* mutations: H481N, L466S, A477D, D471G, H481Y, S464P, S529L, and Q468K.

10.1128/mSphere.00550-17.1 FIG S1  Preferential selection of rifampin resistance *rpoB* mutations. The percentage of predicted Rif^r^ isolates (*y* axis) according to the number of different Rif^r^ mutations (*x* axis) are represented. The eight most frequent mutations predict 93% of the total number of Rif^r^ isolates. Download FIG S1, JPG file, 0.1 MB. Copyright © 2018 Guérillot et al. 2018 Guérillot et al. This content is distributed under the terms of the Creative Commons Attribution 4.0 International license.

### The most frequent rifampin resistance mutations have no negative impact on fitness in rich media and cause cross-resistance to vancomycin and daptomycin.

To test hypotheses generated from our genomic analysis, we reconstructed the eight most prevalent mutations identified by genomic analysis in an isogenic background of the community-acquired (CA)-MRSA clone USA300 NRS384. We also constructed the frequently observed double mutants *rpoB*-H481N-L466S and *rpoB*-H481N-S529L. Whole-genome sequences of all the reconstructed mutants have been determined by Illumina sequencing, and reads were aligned to the wild-type (WT) reference strain (Table S3). Introducing the *rpoB*-H481N mutation led to the emergence of a heterogeneous population of SCV and normal colonies (normal-size colony variant [NCV]) on nonselective HI (heart infusion) agar plates (Fig. S2). Subculturing of the SCV repeatedly produced a mixture of both the SCV and NCV.

10.1128/mSphere.00550-17.2 FIG S2  SCV-NCV mixed colony phenotype associated with the mutation RpoB-H481N. Image of mixed SCV-NCV colony morphology observed on HI agar plate after reconstruction of the dominant Rif^r^ mutation *rpoB*-H481N in the *S. aureus* strain USA300 NRS384. Download FIG S2, JPG file, 0.1 MB. Copyright © 2018 Guérillot et al. 2018 Guérillot et al. This content is distributed under the terms of the Creative Commons Attribution 4.0 International license.

10.1128/mSphere.00550-17.5 TABLE S3  Whole-genome sequencing and SNP calling or reconstructed *rpoB* mutants. Download TABLE S3, XLS file, 0.03 MB. Copyright © 2018 Guérillot et al. 2018 Guérillot et al. This content is distributed under the terms of the Creative Commons Attribution 4.0 International license.

**FIG 5:**
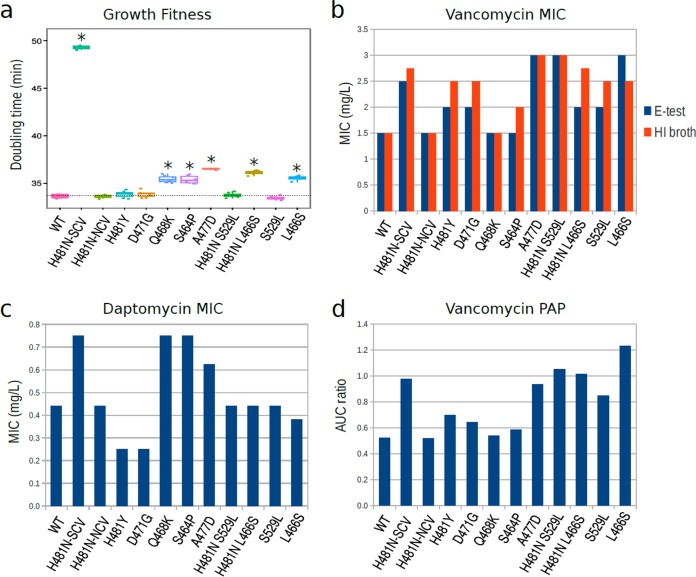
Phenotypic consequences of the most common *rpoB* mutants. (a) *In vitro* fitness of the most frequent Rif^r^ mutations. The growth rate for each bacterial strain was measured at least in triplicate from independent cultures. Each biological replicate is represented by individual data points, and the variability for the doubling time measure for the different strains is depicted with Tukey whisker boxplots. The bottom and top hinges correspond to the first and third quartiles (the 25th and 75th percentiles). RpoB mutants with a median doubling time that differed significantly (*P* < 0.05 by two-tailed Wilcoxon rank sum test) from doubling time for the wild-type strain are indicated with an asterisk. (b) Vancomycin MIC measured by macro Etest and HI broth microdilution. (c) Daptomycin MIC measured by macro Etest. (d) Vancomycin population analysis profile (PAP). Bars represent the measured area under the curve (AUC) ratio of each mutant versus the reference hVISA strain Mu3 (ATCC 700698). Etest and broth microdilution MIC values were measured by two independent experiments (biological replicates) and the plates were read in a blind manner (researchers reading the plates did not know which strains were being read). The histograms represent average MIC values. For PAP, single cultures were diluted and plated two times at 10^−3^ and 10^−6^ (technical replicates). The average CFU on BHI plate with vancomycin at 0, 0.5, 1, 1.5, 2, 2.5, 3, and 4 μg per ml were used to calculate AUC. All measured values are reported in Table S4 in the supplemental material.

The fate of a resistance mutation in pathogen populations is determined in part by its impact on fitness. Mutations that incur little or no fitness cost are more likely to persist in the absence of antibiotic treatment (37). To test the hypothesis that the relative abundance and number of selection events of preferential Rif^r^ mutations are reflected by a reduced fitness cost, we performed an *in vitro* fitness assay with the reconstructed *rpoB* mutants. We compared the maximum growth rates of the 10 reconstructed *rpoB* mutants against that of the wild-type isolate. As the *rpoB*-H481N mutant is associated with a heterogeneous population of SCV and NCV, we measured the growth rates of both colony variants. Small increases in doubling times were observed for five mutants (L466S, H481N-L466S, S464P, Q468K, and A477D) (Fig. 5a). No significant difference in doubling time was observed compared to the wild-type strain for the two most frequently encountered Rif^r^ *rpoB* mutations, H481N-NCV and H481Y, and three other preferential Rif^r^ mutations (S529L, H481N-S529L, and D471G). As expected, the H481N-SCV mutant showed a significant increase in doubling time (Fig. 5a).

10.1128/mSphere.00550-17.6 TABLE S4  Resistance to rifampin, vancomycin, and daptomycin of *rpoB* mutants. Download TABLE S4, XLS file, 0.03 MB. Copyright © 2018 Guérillot et al. 2018 Guérillot et al. This content is distributed under the terms of the Creative Commons Attribution 4.0 International license.

Among Rif^r^ preferential mutations, A477D, was previously identified as promoting decreased susceptibility to both vancomycin and daptomycin (17, 27) and mutations H481Y and Q468K have been associated with decreased susceptibility to vancomycin and daptomycin, respectively (16, 17). Despite these observations, only the *rpoB*-H481Y mutation has been confirmed by genetic reconstruction to confer vancomycin cross-resistance (16). To accurately evaluate the effects of common Rif^r^ mutations on vancomycin and daptomycin cross-resistance, we measured the MIC of our reconstructed *rpoB* mutants (Fig. 5b to d and Table S4). Using three different MIC methods, we confirmed that mutations H481Y and A477D increased vancomycin MIC. We found that six new *rpoB* alleles conferred decreased susceptibility to vancomycin (H481N, L466S, S529L, H481N-L466S, H481N-S529L, and D471G). We also confirmed modest but nonetheless significant increased daptomycin MICs for a range of mutants, including *rpoB*-Q468K, *rpoB*-A477D, *rpoB*-H481N SCV, and *rpoB*-S464P. Thus, all the most frequent *rpoB* alleles were associated with decreased susceptibility to vancomycin and/or daptomycin.

### Rifampin use is compromising susceptibility to last-line antibiotics.

**FIG 6:**
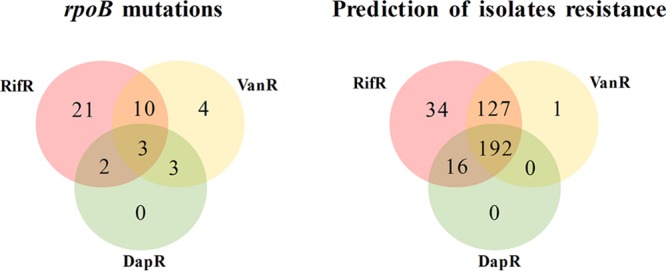
Prediction of cross-resistance among rifampin-resistant isolates. The Venn diagram on the left shows known RpoB mutations that have been associated with rifampin resistance (red), vancomycin reduced susceptibility (yellow), and daptomycin reduced susceptibility (green). Rifampin resistance, vancomycin resistance, and daptomycin resistance are indicated by RifR, VanR, and DapR, respectively, in the figure. The number of resistance mutations is indicated in each circle, and the number of mutations associated with cross-resistance is indicated in the intersection of the circles. The right diagram shows the observed number of isolates harboring these mutations among the 7,099 isolates analyzed and depicts the high number of isolates predicted to be resistant to vancomycin and daptomycin among the Rif^r^ isolates.

We showed above that the most prevalent and frequently selected Rif^r^ mutations all have an impact on daptomycin and vancomycin MIC. We then used these laboratory findings to infer the impact of these mutations on a global scale. We compiled a Venn diagram with all *rpoB* mutations and their associated resistance to rifampin or cross-resistance phenotype to vancomycin and/or daptomycin (Fig. 6). We then conducted the same analysis but used the publicly available *S. aureus* genome sequences with these mutations. This analysis predicted that 86% (319/369) of Rif^r^ isolates should exhibit decreased susceptibility to vancomycin, 56% (208/369) to daptomycin and 52% (192/369) to both vancomycin and daptomycin (Fig. 6). This suggests that *S. aureus* rifampin resistance (a consequence of rifampin usage) is leading to decreased susceptibility to the last-line antibiotics vancomycin and daptomycin globally.

## DISCUSSION

This study has shown that preferentially selected *rpoB* mutations promote clinically relevant phenotypic plasticity through the generation of a subpopulation of SCVs, heteroresistance to vancomycin, and reduced susceptibility to daptomycin. Among a global collection of *S. aureus* isolates, rifampin usage appears to have led to the emergence of multiple stable Rif^r^ lineages that are therefore predicted to be associated with increased risk of therapeutic failure. Given previous work demonstrating a clear link between *rpoB* Rif^r^ mutations in *S. aureus* and alterations in host-pathogen interactions promoting persistent infection (15, 27, 28), our study highlights the global impact of antimicrobial selective pressure in driving antimicrobial cross-resistance and persistence in difficult-to-treat infections.

Our analyses highlight several concerns regarding Rif^r^ selection in *S. aureus*, indicating that specific approaches are required to address this problem on a global scale. First, Rif^r^ mutations are common among clinical *S. aureus* isolates, especially CC5 and CC8 MRSA isolates of human origin, and in isolates from the Asia-Pacific region and South America. Second, some Rif^r^ mutations are not associated with an identified laboratory fitness cost, and Rif^r^ clones successfully spread and are transmitted at large geographic scales and can persist for decades. Selection of Rif^r^ preferentially targets a small number of mutations that account for most of the resistant phenotypes. Critically, these selected mutations can have broader negative therapeutic consequences related to their pleiotropic effect on resistance to vancomycin and daptomycin, emergence of SCV, and association with immune evasion. A tempting hypothesis is that when selecting for Rif^r^, *S. aureus* clones carrying specific *rpoB* mutations conferring lower fitness cost and adaptive positive pleiotropic effects, like resistance to other last-line antibiotics, are more likely to be successful and can lead to stable conversion of rifampin-resistant lineages. Notably, four of the most frequent *rpoB* mutations identified here confer only low-level Rif^r^, equal to or below the Clinical and Laboratory Standards Institute (CLSI) breakpoint (MIC ≥ 4 mg/liter) (see Table S4 in the supplemental material), suggesting that these isolates would not be detected as resistant in laboratories using these antibiotic susceptibility criteria. In contrast, the current European Committee on Antimicrobial Susceptibility Testing (EUCAST) breakpoint (MIC ≥ 0.06 mg/liter) would detect all isolates containing these mutations.

We argue here that decreased susceptibilities to rifampin, vancomycin, and daptomycin are coselected at the *S. aureus* global population level through selection of *rpoB* variants. Our analysis indicates that rifampin usage and not vancomycin or daptomycin usage is the main selective pressure responsible for this coselection through fixation of specific Rif^r^ *rpoB* mutations. Interestingly, we observed a higher prevalence of Rif^r^ isolates (Fig. 1c), as well as repeated examples of successful clonal expansion of Rif^r^ clones (Fig. 4), in *S. aureus* lineages CC8 (ST239) and CC5 (ST5) which also corresponded to the lineages that have a higher median vancomycin MIC (5). These correlations strongly support the idea that Rif^r^ selection is a major driving force toward decreased susceptibility to vancomycin. Our observations are corroborated by two recent genome-wide association studies (24, 25). These two studies found that the strongest genetic marker for the hVISA phenotype is the *rpoB* residue 481, which we found here to represent the most common Rif^r^ mutation, *rpoB*-H481N, and also the two most frequently selected Rif^r^ mutations (*rpoB*-H481N and *rpoB*-H481Y). Taken together with previous studies, our data demonstrate for the first time by genetic reconstruction and phenotype testing the role played by commonly selected Rif^r^ mutations in promoting cross-resistance to vancomycin and daptomycin.

An intriguing finding of this study was the identification of the most common Rif^r^ mutation *rpoB*-H481N as a mutation promoting an unstable SCV phenotype. *S. aureus* SCVs are well-known to be associated with chronic, recurrent, and antibiotic-resistant infections (38, 39). Numerous studies have investigated the role of SCV on persistent infection and its mechanism by using mutants impaired in important metabolic pathways such as the electron transport chain (39). It has previously been reported that the RpoB H481Y mutation can be associated with a remarkably persistent *S. aureus* infection (15, 36), and it was demonstrated that this specific mutation contributed to the SCV phenotype and persistent infection through immune evasion. While the molecular mechanisms underpinning the mixed colony morphotypes identified in the H481N mutant in this study are not yet defined, the association of this phenotype with this mutation further enhances the potential difficulty in controlling *S. aureus* infections in strains containing this mutation. Moreover, we identified among preferential Rif^r^ mutations three other RpoB mutations that have been phenotypically demonstrated as promoting persistent infection: H481Y, A477D, and S464P (15, 27, 28).

As epidemiological studies are moving toward genomic surveillance, global and large-scale genomics-based epidemiological analysis promise to become a powerful tool to efficiently track and prevent antimicrobial resistance. By carefully compiling current knowledge on mutational resistance, we show here that large-scale phylogenetic analysis can provide valuable information about the epidemiology and persistence of antimicrobial resistance. By tracking the occurrence and frequency of acquisition and the phylogenetic persistence of Rif^r^ mutations, we were able to identify key mutations that deserve the focus of both epidemiological surveillance and phenotypic characterization. Furthermore, as similar resistance can be conferred by different mutations that can be associated with different therapeutic risks, characterization at the single nucleotide level of resistance has the potential to optimize patient treatment in the future. It should be noted that our analysis is limited by the use of a convenient collection of publicly available genomes that represents a biased sample of the worldwide distribution of *S. aureus* isolates, for example, the overrepresentation of MRSA isolates in the collection. Therefore, no definitive epidemiological conclusion can be drawn from the data.

Despite numerous studies that identified a wide range of *rpoB* mutations arising after exposure to different antimicrobials and antimicrobials other than rifampin, almost none were found within the broad panel of clinical isolates studied here. Our study highlights the value of contextualizing *in vitro* studies investigating resistance mutation with population and evolutionary genomic studies to enable the identification of clinically relevant resistance mutations and their underlying mechanisms. Here, we demonstrate the power of this approach through large-scale phylogeny coupled with evolutionary convergent analysis to identify key persistent resistance mutations or other potential adaptive mutations that should be examined more thoroughly.

Further investigation will be needed to decipher the underlying mechanisms leading to pleiotropic phenotypic change associated with each particular Rif^r^ mutation and to identify drivers of higher rates of resistance in certain regions. From existing knowledge, we suggest that rifampin should be used with care, as we observed that the majority of selected Rif^r^ clones are associated with cross-resistance to last-line antibiotics used to treat MRSA and/or phenotypes that can favor persistence. Failure to detect clones harboring *rpoB* mutations that can result in cross-resistance to other antimicrobials could be addressed by harmonizing international susceptibility breakpoints at the lower level. This recommendation together with appropriate rifampin prescribing can be effective in reducing the fixation and spread of these adverse mutations.

## MATERIALS AND METHODS

### Genomic data.

To obtain a global representation of *S. aureus* diversity, we downloaded 7,364 *S. aureus* genomes comprising all available *S. aureus* assemblies from the PATRIC database (2,841 in August 2015) (40), all NCBI genome assemblies not present in the PATRIC database (2,845 in February 2016), and the Sequence Read Archive (SRA), originating from five different *S. aureus* sequencing projects (PRJEB5261, 925 strains; PRJNA239000, 147; PRJNA239001, 75; PRJNA252378, 398; PRJNA275322, 133). Metadata were collected from the PATRIC database and the NCBI BioProject database. All sequence files were processed with Snippy 2.9 (T. Seemann; https://github.com/tseemann/snippy) and mapped against a PacBio finished reference genome of *S. aureus* USA300 strain NRS384. High-confidence variants were called by removing aligned reads having mapping quality below 60 and requiring a minimum depth of 20 reads with at least 90% of reads supporting the variant. To remove low-quality genome sequences, we kept only the genomes where 80% or more bases were aligned to the reference genome and strains with ambiguous *rpoB* alignment were excluded. In total, we kept 7,099 *S. aureus* genomes for downstream analysis. Genomes associated with metadata indicating a human origin were defined as clinical isolates (6,259 isolates). Multilocus sequence type (MLST) was determined *in silico* by MLST (v2.6) (T. Seemann; https://github.com/tseemann/mlst). MRSA/MSSA was inferred by detecting the *mecA* gene by BLAST using abricate (v0.3) (T. Seemann; https://github.com/tseemann/abricate).

### Phylogenetic analysis.

The complete alignment of the 7,099 genomes was filtered with SNP sites (41) (v2.3.2) to remove monomorphic sites and then trimmed with Trimal (42) (v1.4.rev15) to retain only alignment positions with less than 2% gaps or ambiguous positions. Maximum likelihood phylogenies were inferred with FastTree (41) (v2.1.8) and with RAxML (43) (v8.2.9) using the generalized time-reversible (GTR) model with optimization of substitution rates and gamma distribution to account for among site rate heterogeneity. For RAxML phylogenies, three alternative runs were performed on distinct starting trees using the command: raxmlHPC-PTHREADS-AVX -s infile.aln -n outfile.tree -m GTRCATX -V -T 25 -N 3 -p 42. To identify homoplastic mutations (convergent mutations) and Rif^r^ monophyletic clades, clade branch support was computed with FastTree. Monophyletic clades were confirmed on both inferred phylogenies. To further confirm monophyly, clonal complex (CC)-specific core genome SNP alignments were computed, and the CC-specific phylogenies were inferred with FastTree.

### Convergent mutation analysis.

Convergent mutation analysis was performed in R using the *phylobase* and *ape* packages (44). Trees were imported into R and were traversed node by node from the tip to the root starting from each tip/strain coding for a mutated *rpoB* allele. At each node if all descendants of the node shared the same *rpoB* mutation, tree traversal continued toward a node closer to the root to potentially enlarge the monophyletic clade. If all the descendants of a node did not share the same *rpoB* mutation, tree traversal was stopped and started from another tip/strain with the same *rpoB* mutation. This was applied to all identified* rpoB* mutations. For each mutation, we counted the numbers of independent monophyletic clades, which represents an approximation of the number of independent acquisitions of a specific mutation. Trees were plotted and annotated using the *ggtree* and *ggplot2* R packages (45, 46).

### Construction of *rpoB* mutants by allelic exchange.

Allelic exchange experiments were performed using shuttle vector pIMAY-Z as previously described (47) with modifications. Briefly, full-length *rpoB* sequences containing the 10 different *rpoB* alleles were reconstructed by performing PCR overlap extension with Phusion high-fidelity DNA polymerase (New England Biolabs) and introducing *rpoB* codon mutations into the primer tails (see Table S5 in the supplemental material for the primers). Purified *rpoB* amplicons were then joined with pIMAY-Z using seamless ligation cloning extract (SLiCE) cloning (48) and subcloned into *E. coli* IM08B strains (47) to allow CC8-like methylation of the plasmid and avoid the *S. aureus* restriction barrier. The presence of a cloned *rpoB* insert in pIMAY-Z plasmid was then confirmed by colony PCR using primers pIMAY-Z-MCSF and pIMAY-Z-MCSR. Purified plasmids were then electroporated into *S. aureus* USA300 strain NRS384 and plated on heart infusion (HI) agar supplemented with chloramphenicol (Cm) at 10 mg/liter and X-Gal (5-bromo-4-chloro-3-indolyl-β-d-galactopyranoside; Melford) at 100 mg/liter and grown for 48 h at 30°C. Blue colonies were picked and grown on HI broth at 37°C without Cm selection pressure overnight to allow loss of the pIMAY-Z thermosensitive plasmid. Double crossover leading to allelic replacement of the wild type with the desired Rif^r^ *rpoB* alleles were directly selected by plating culture on HI agar plates supplemented with 0.06 mg/liter of rifampin (RIF). Rif^r^ and chloramphenicol-sensitive colonies arising at a frequency higher than 10^−3^ were considered potentially positive clones for allelic exchange, as spontaneous Rif^r^ arise at a much lower frequency of ~10^−6^ in the wild-type strain (data not shown). The clones were then colony purified on HI agar plates before glycerol storage and genomic DNA was extracted. To validate the allelic exchange procedure, the whole-genome sequence of all reconstructed strains was determined with the Illumina Miseq or Nextseq platforms, using Nextera XT paired-end libraries (300-bp or 150-bp paired ends, respectively). To ensure that no additional mutations were introduced during the allelic exchange procedure, reads of all mutant strains were mapped to the reference NRS384 “wild type” genome using Snippy (v 2.9). The SNP profile for each mutant is presented in Table S3.

10.1128/mSphere.00550-17.7 TABLE S5  Primers used in this study. Download TABLE S5, XLS file, 0.03 MB. Copyright © 2018 Guérillot et al. 2018 Guérillot et al. This content is distributed under the terms of the Creative Commons Attribution 4.0 International license.

### Fitness assays.

Single colonies of *S. aureus* were resuspended in phosphate-buffered saline (PBS) at a McFarland standard of 0.2. Five microliters of bacterial suspension was inoculated in 200 µl of HI broth in 96-well plates. Bacteria were incubated at 37°C with agitation, and the optical density at 600 nm (OD_600_) was measured every 15 min for 24 h using an EnSight multimode plate reader (PerkinElmer). The maximum growth rate and doubling time were determined by fitting local regression over intervals of 1 h on growth curve data points and by taking the maximum value of the fitted derivative using the R package *cellGrowth* (49). The growth rate for each bacterial strain was measured at least in triplicate from independent cultures. The *rpoB* mutant growth rates were compared with the growth rate of the wild-type strain NRS384 to measure relative growth fitness.

### Antibiotic susceptibility testing.

Standard Etests (bioMérieux) were performed according to manufacturer’s instructions. Macro Etests were performed as previously described (4). Vancomycin population analysis profiles (PAPs) were performed as previously described (50) and by allowing 48 h of growth in order to isolate slow-growing VISA subpopulations. Broth cultures were performed in the wells of 96-well plates in 200 µl of HI broth alone and supplemented with 0.5, 1, 1.5, 2, 2.5, or 3 mg/liter of vancomycin. To avoid batch effects, all mutant strains and wild-type strain were tested with the same batch of HI broth and HI broth plus vancomycin solutions, and growth was monitored in the same 96-well plate. Overnight HI broth cultures of the strains to be tested were adjusted to an OD_600_ of 5, and 2 µl was inoculated into each well. Growth was then immediately monitored at OD_600_ for 36 h using a PerkinElmer EnSight plate reader.
