# Supplementary figures and images for: Convergent Evolution Driven by Rifampin Exacerbates the Global Burden of Drug-Resistant Staphylococcus aureus
Source: mSphere. 2018 Jan 24;3(1):e00550-17. doi: 10.1128/mSphere.00550-17 (PMC5784246; doi:10.1128/mSphere.00550-17)

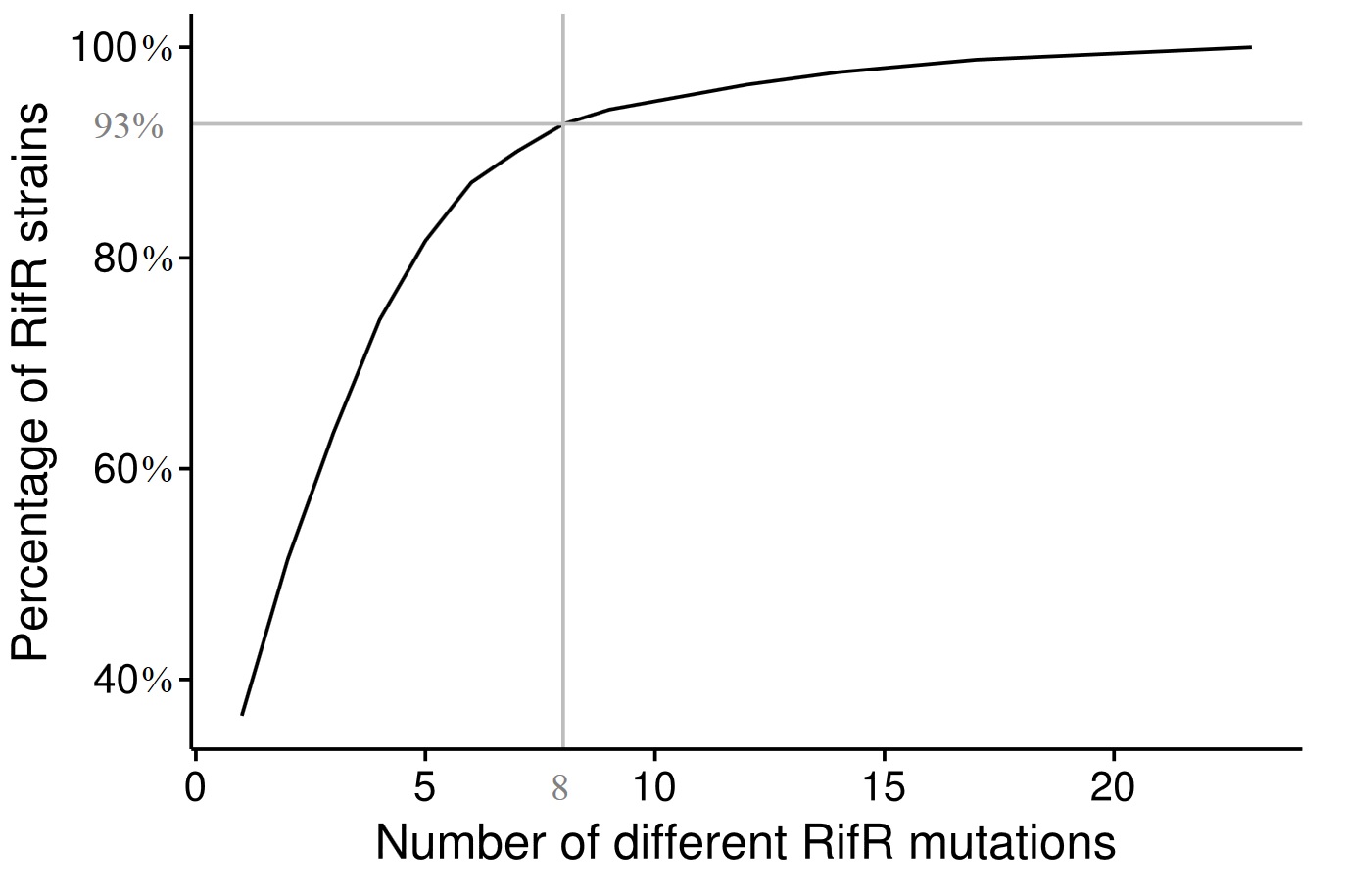

Supplement: FIG S1 [file sph001182461sf1.jpg]

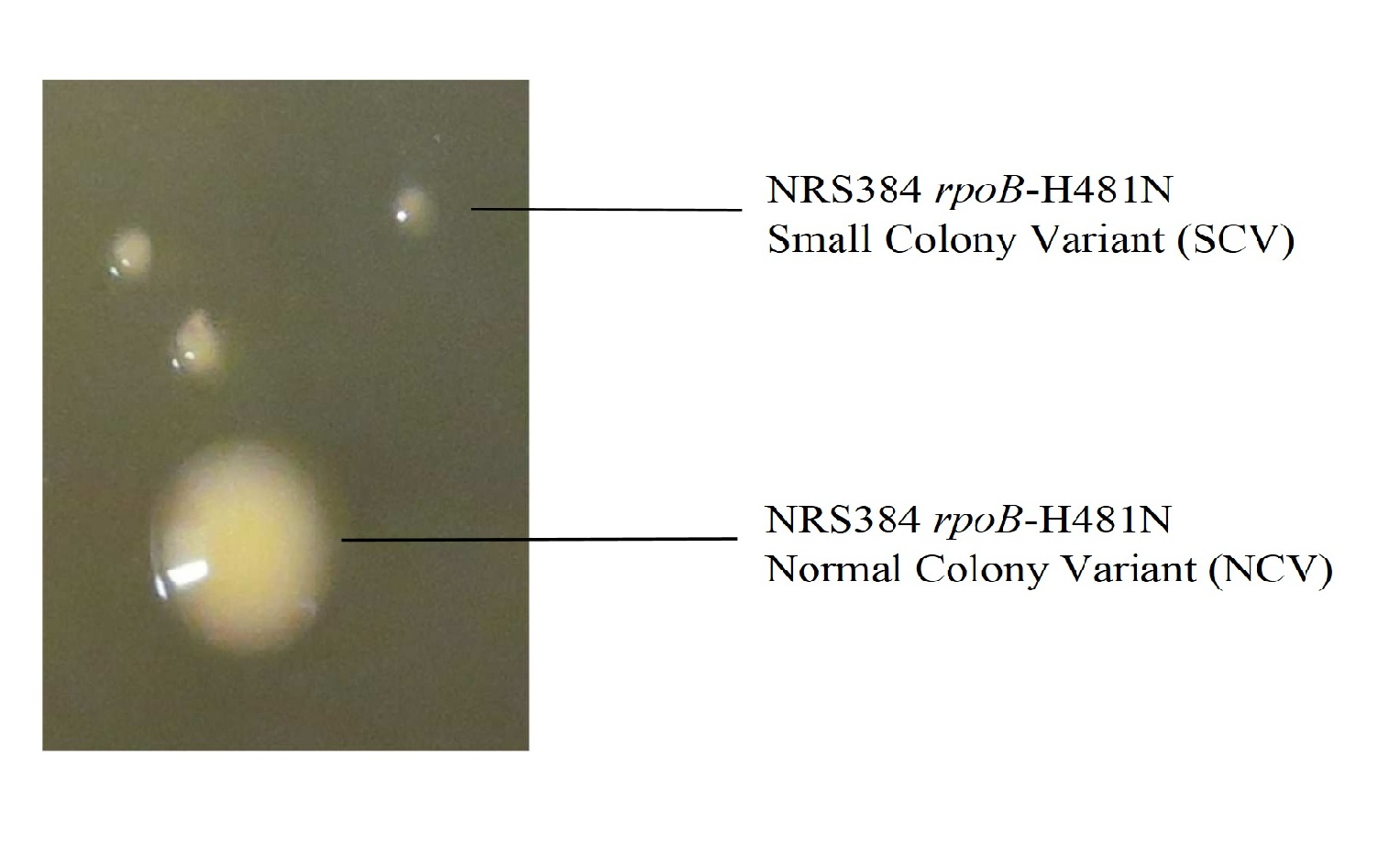

Supplement: FIG S2 [file sph001182461sf2.jpg]
